# Multiple sound sources localization in free field using acoustic vector sensor

**DOI:** 10.1007/s11042-013-1549-y

**Published:** 2013-06-21

**Authors:** Józef Kotus

**Affiliations:** Multimedia Systems Department, Gdansk University of Technology, Narutowicza 11-12, 80-233 Gdansk, Poland

**Keywords:** Sound detection, Sound source localization, Audio surveillance

## Abstract

Method and preliminary results of multiple sound sources localization in free field using the acoustic vector sensor were presented in this study. Direction of arrival (DOA) for considered source was determined based on sound intensity method supported by Fourier analysis. Obtained spectrum components for considered signal allowed to determine the DOA value for the particular frequency independently. The accuracy of the developed and practically implemented algorithm was evaluated on the basis of laboratory tests. Both synthetic acoustic signals (pure tones and noises) and real sounds were used during the measurements. Real signals had the same or different energy distribution both on time and frequency domain. The setup of the experiment and obtained results were described in details in the text. Taking the obtained results into consideration is important to emphasize that the localization of the multiple sound sources using single acoustic vector sensor is possible. The localization accuracy was the best for signals which spectral energy distribution was different.

## Introduction

Audio source localization using an array of sensors is a rich topic which has interested many signal processing researchers for many years. Applications e.g. include speaker location discovering in a teleconference, event detection and tracking, robot movement in an unknown environment, etc. A lot of techniques were proposed and can be found in the literature. Currently considered issues focuses on improving the well-known techniques, or the search for new solutions. For example Blandin et al. introduced several multi-source TDOA estimation methods based on angular spectra and clustering. They consider the problem of estimating the time differences of arrival (TDOAs) of two or more sources for a given pair of sensors in a reverberant environment. They evaluated his own method and five most popular state-of-the-art methods on 1482 different configurations. They conclude that the tested techniques that have been successful for anechoic TDOA estimation and audio source separation bring a little or no improvement for reverberant TDOA estimation. So that more specific approaches are needed to solve this problem in the future [3]. Pavlidi et al. proposed a novel real-time adaptative localization approach for multiple sources using a 8 microphone circular array, in order to suppress the localization ambiguities faced with linear arrays, and assuming a weak sound source sparsity which is derived from blind source separation methods [16]. They conclude that his method performs very well both in simulations and in real conditions at online processing. Different approach to the sound source localization was proposed by Stanacevic and Cauwenberghs. They used a gradient flow technique for localization of an acoustic source using miniature microphone arrays by relating temporal and spatial gradients of the impinging source signal [22].

This work base on acoustic data obtained by matrix of microphones in different configurations. In this paper author applied 3D Acoustic Vector Sensor to multiple sound sources localization. Acoustic vector sensors were first applied to acoustic source localization in the air by Raangs et al. in 2002, who used measured sound intensity vector to localize a single monopole source [20]. A more recent development is the application of acoustic vector sensors to the problem of localizing multiple sources in the far field. In 2009, Basten et al. applied the MUSIC method to localize up to two sources using a single acoustic vector sensor [1]. In the same year Wind et al. applied the same method to localize up to four sources using two acoustic vector sensors [23, 24].

In author previous works the sound source localization methods based on sound intensity computed in time domain were presented [5, 9–15, 23, 24]. Those techniques worked with broadband signals received from multichannel acoustic vector sensor [6]. Single, dominant sound source was localized properly by means of those methods [5, 6, 11–15]. When more than one sound source produced the acoustic energy simultaneously, determination their positions was very difficult. Quite different approach to multiple sound sources localization online using the acoustic vector sensor was presented in this study [7, 8]. Online processing means that the algorithm deliver the localization results with very small time delay, around 0.1 [s]. Term of the multiple sound sources in this research means that two sources produced the acoustic energy simultaneously from different directions. Main differences depends on computation the sound intensity components in the frequency domain. Fast Fourier Transform was applied for this purpose. Obtained spectrum coefficients for considered signal allowed to determine the DOA values for the particular frequency independently. Developed algorithm was designed to analyze the acoustic signals in real time. It was presented in details in Section 2. Due to the computation of sound intensity for particular frequency independently, the method should work properly for signals which are different in the frequency domain. It is important to emphasize that localization process of sound source takes into consideration the dynamic of acoustic energy emission. It means that even if the particular sound sources have the same average spectral energy distributions they can still be localized properly. It will took place if they will produce the energy in different parts of time (sound sources should be incoherent). Applied method, used signals and organization of measurement tests were described in details in Section 4. Obtained results were presented in Section 5.

## Multiple sound sources localization algorithm

**Fig. 1:**
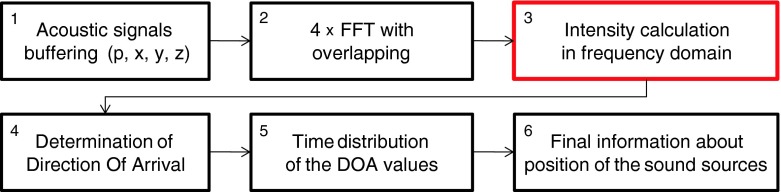
The block diagram of the proposed algorithm. *Red rectangle* indicate the most important part of the developed method

Proposed, implemented and practically evaluated algorithm to multiple sound sources localization is based on calculation sound intensity level in frequency domain. It is a quite different approach in opposite to methods which rely on sound intensity calculation in the time domain. The block diagram of the algorithm was presented in Fig. 1. Calculation process was divided into six functionally different steps. Particular phases were described in details below.

The applied multichannel acoustic vector sensor produces the following signals: sound pressure *p* and three orthogonal particle velocity components *u*_*x*_, *u*_*y*_, *u*_*z*_. It is important to emphasize that various transducers topologies can be used to measure the acoustic velocity directly or indirectly and such kind of sensors could be also applied in the proposed algorithm [4]. In the first step each signals were buffered and prepared to FFT calculation. The Hanning window was applied in this case [21]. Next the 4096 point FFT calculation for each signals were performed, sampling frequency was equal to 48 kHz (frequency resolution: 11.7 Hz). Such parameters provide sufficient spectral resolution. It is important to emphasize that the proper overlapping technique is crucial for the next steps. The overlap degree from 0 % to 75 % was tested (it was explained and illustrated in the results section). It means that in the next FFT frame from 4096 to 1024 samples were new. On the other hand for 4096 signal samples from 1 to 4 FFT frames were calculated. The FFT calculation were performed for each acoustic components separately. In block 3 the sound intensity in frequency domain was computed. Intensity component for *x* direction and *i*-th frequencies was defined as

1 $$ {I}_x(i)={X}_p(i)\cdot \overline{X_{ux}(i)} $$

where:

*X*_*p*_(*i*): coefficients of complex spectrum for *i*-th frequencies for acoustic pressure signal
$$ \overline{X_{ux}(i)} $$
conjugated spectrum coefficients for particle velocity into *x* direction.

Intensity components for *y* and *z* direction were computed in the same way. The final intensity vector was given by equation [2]

2 $$ \overrightarrow{I}={I}_x{\overrightarrow{e}}_x+{I}_y{\overrightarrow{e}}_y+{I}_z{\overrightarrow{e}}_{{}_z} $$

It is the most important part of whole algorithm. The direction of arrival for particular frequency was obtained as a result of this block (with given spectral resolution, 11.7 Hz in this case). After that the direction of arrival for each frequencies were determined. The values of azimuth and elevation angles were calculated based on transformation from Cartesian to spherical coordinate system. Obtained DOA values were used to compute its time distribution. The time distribution collects the intensity values for given direction with 1 degree quantization. To reduce the noise level in the computed time distribution characteristics not all intensity vectors were used. During the computation process, the noise floor (the average magnitude values of acoustic pressure obtained in step 2) for particular frequencies was calculated. The part of the signal between the end of first reference sequence and beginning of the fist acoustic event of the measurement session was used for this purpose (the measuring scenarios were presented in details in the paragraph 3.3). Only the intensity vectors which value exceeded the noise floor increased by the defined threshold were used to accumulate the time distribution for given direction. In presented algorithm the threshold was equal to 10 dB (for this value the noise influence can be neglected, moreover in future practical implementation the threshold value could be specified by the user). In the accumulating process the value of the intensity vector was additionally used. In fact the time distribution indicate the total value of intensity vectors which occurred for given direction in considered time period. The maximum value observed in the time distribution characteristics indicate the position of the sound source. When more than one sound source was present in the acoustic field in considered time period, the additional maximum value on time distribution characteristic can occur. At the end of the calculation process (block 6), the final information about the position of particular sound sources position was indicated. The final time distribution of DOA values was smooth by means of weighted moving average. Angle values for particular peaks were obtained using local difference calculation. The difference between the actual and next DOA value was computed. When the difference changes the sign from positive to negative, the local maximum should occur. If the difference is greater than assumed threshold it means that it indicate the possible position of sound source.

## Evaluation of the proposed algorithm

For simplify and reduce the complexity of the description of localization accuracy only azimuth angle was taken into consideration. Elevation angle was neglected. The sound source localization accuracy (α_err_) was defined in that case as a difference between the computed direction of arrival angle (α_AVS_) and Ground Truth angle value (α_GT_) (it indicate the real position of the sound source) for considered acoustic event. This parameter was given by the equation

3 $$ {\alpha}_{\mathrm{err}}={\alpha}_{\mathrm{AVS}}-{\alpha}_{\mathrm{GT}} $$

The evaluation process relied on determination the α_err_ value for every sound source. To proper determination of this parameter both α_AVS_ and α_GT_ should be known. The proposed sound sources localization algorithm returns the result as a value of the angular direction of arrival for particular sound sources (α_AVS_). The angular values and localization accuracy are expressed in degrees. The multichannel loudspeaker system placed in the anechoic chamber was used to simulate the sound sources, therefore the α_GT_ values were easy to determine. The prepared measurement system, used test signals and realized scenarios were described in this section.

### Measurement system

**Fig. 2:**
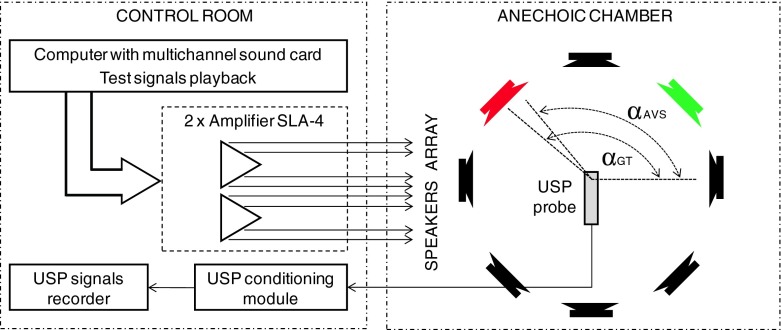
Setup of measurement system, *red* and *green color* of the loudspeakers presents one of the scenario, when two different sounds were played

**Fig. 3:**
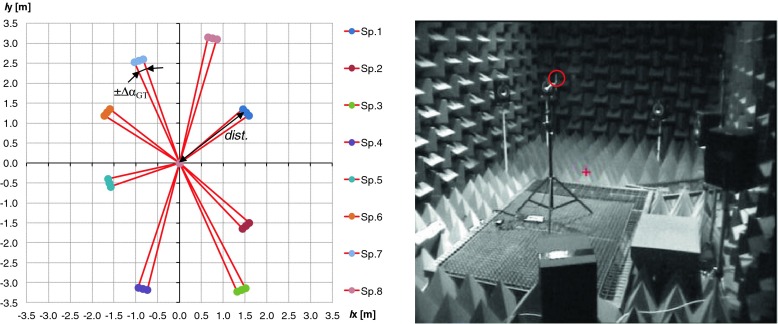
*Left* – details positions of particular loudspeakers. *Right* picture presents the interior of anechoic chamber, *red circle* indicate the position of used acoustic vector sensor (USP probe)

Setup of the measurement equipment employed in the experiment is presented in Fig. 2. Placement of speakers and angles (α) between them and the USP probe were presented in Figs. 2 and 3.

In the anechoic chamber 8 REVEAL 601p speakers [19], an USP probe were installed. The USP probe was fixed about 1.5 m above the floor. In the control room a PC computer with Marc 8 Multichannel audio interface was used to generate test signals. Signals from the USP probe were recorded using MAYA44 USB audio interface [17]. Two SLA-4 type 4-channel amplifiers were employed to power the speakers [18]. The width of the speakers (0.21 [m]) was also measured and illustrated in Fig. 3 (Left). This value was used to calculate Δα_GT_ parameter. Real angle values between the speakers and USP Probe were used as a reference data during the evaluation process (see α_GT_ in Eq. 3). Details positions of particular loudspeakers and hardware setup were presented in Fig. 3. Right picture presents the interior of anechoic chamber, red circle indicate the position of used acoustic vector sensor (USP probe). Used loudspeaker system can also be noticed. The anechoic chamber simulates the conditions of the free acoustic field. Reflections coming from different surfaces can be neglected. It is important to emphasize two essential things. First, the distance between the USP probe and loudspeaker is rather small. The dimensions of the sound source (width of the used loudspeakers) was taken into consideration in the localization accuracy estimation. Second, the used loudspeakers only simulate the real sound source. In practice, the sound source that produce considered type of acoustic signal can have different dimensions. That facts can be important to estimate the correctness of the sound sources localization in real conditions.

The detailed values of ground truth data of DOA for particular loudspeakers and results obtained for references signals presented at the beginning and end of given measurement scenario were presented in Table 1. The used measurement scenarios and calibration procedure were described in details in the next sections.

**Table 1:** Ground Truth data, distance to the AVS sensor (in [m]) and Δα for each speaker

Sp. No.	1	2	3	4	5	6	7	8
α_GT_	40	314	294	255	197	143	110	77
*dist.*	1.984	2.182	3.476	3.259	1.687	2.098	2.737	3.223
Δα_GT_	±3.0	±2.7	±1.7	±1.8	±3.5	±2.9	±2.2	±1.9

### Calibration procedure

**Fig. 4:**
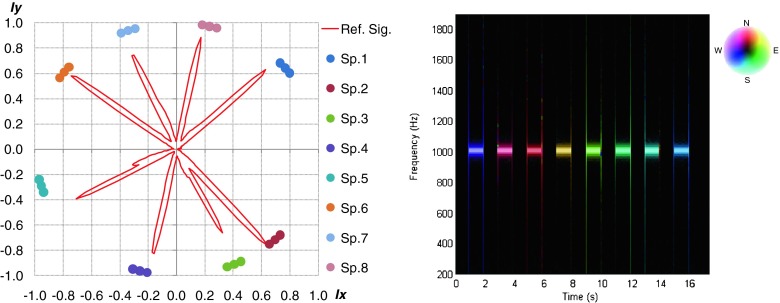
The localization of the reference signals emitted in sequence by particular loudspeakers

For every measurement session two reference sequence were used, before (α_AVSRef.1._) and after (α_AVS Ref.2._) the test signals presentation. Pure tone about 1 kHz frequency and one second duration time was played by particular loudspeakers sequentially. This signal was used to prepare and calibrate the whole measurement system. Hardware setup and measurement session were proper when the localization results of the particular loudspeakers for both calibration sequences were the same. The localization results of particular reference signals emitted in sequence by particular loudspeakers were presented in Fig. 4. Spectrogram DOA for reference sequence was also depicted. For better presentation the angular data positions of the loudspeakers were normalized.

The detailed values of ground truth data of DOA for particular loudspeakers and results obtained for references signals presented at the beginning and end of all measurement scenarios were presented in Table 2. The values obtained during both reference sequences were the same. It means that the hardware setup was proper during whole experiment.

**Table 2:** Ground Truth data and αerr obtained for both reference signals

Sp. No.	1	2	3	4	5	6	7	8
α_GT_	40	314	294	255	197	143	110	77
α_AVS Ref.1._	43	309	294	251	205	132	110	79
α_AVS Ref.2._	43	309	294	251	205	132	110	79
α_err_.	−3	5	0	4	−8	11	0	−3

### Test signals and measurement scenarios

**Fig. 5:**
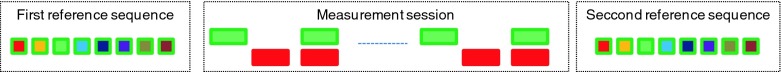
Time organization of measurement scenarios

Both synthetic acoustic signals (such as pure tones and narrow band pink noises) and real sounds were used during the measurements. Pure tones has the following frequencies: 250 Hz, 500 Hz, 800 Hz, 1000 Hz, 1250 Hz, 2000 Hz, 4000 Hz. Noises signals were prepared on the basis of pink noise broadband signal filtrated by means one third octave band filters. Center frequencies of particular filters were the same like for tonal signals. Signal amplitudes have been normalized therefore they had the same acoustic energy. Selected real signals used during the tests were different in the frequency and time domain. Exemplary recordings several type of sound sources such like: speech, scream, car horn, shot and broken glass were prepared and applied during the tests. The main assumption of the prepared measurement scenarios was simultaneous presentation signals which have the same or different energy distribution both in time and frequency domain. Main aim of such scenarios was evaluation of localization accuracy as a function of characteristic of the sound source. For this purpose during one session the particular sound source was used two times. First the single sound source from one direction was played, next second sound source played from other direction and finally both sound sources were played simultaneously from their directions. The time organization of the measurement scenarios was presented in Fig. 5.

**Fig. 6:**
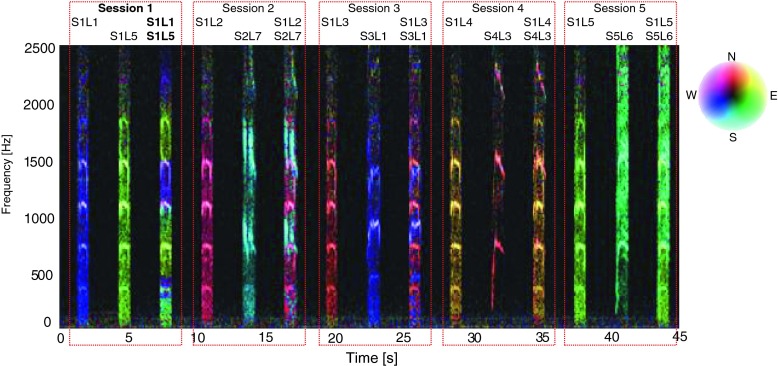
Example of the frequency characteristic of selected test signals (scream) and measure scenario (*dotted red rectangles*). During session 1 identical sound signals were presented from different directions (coherent sound sources). In other sessions different scream signals were used

For such arrangement of the sound source reproduction, the localization accuracy of single and multiple sound sources can be determined. Another parameter which was taken into consideration was the cohesion of sound sources. It was verified by reproduction different or the identical sounds from given directions. For identical sound sources we do not notice any differences between the sound sources both on time and frequency domain. It means that proposed algorithm in such conditions cannot work properly. The example frequency characteristic of selected test signals and measure scenario were presented in Fig. 6.

All calculations and experiments were performed using ASUS B50A computer (Intel Core 2 Duo 2.2 GHz CPU with 3 GB RAM memory) with 32-bit Windows Vista operating system.

## Results

### Calculation of time distribution of DOA values

**Fig. 7:**
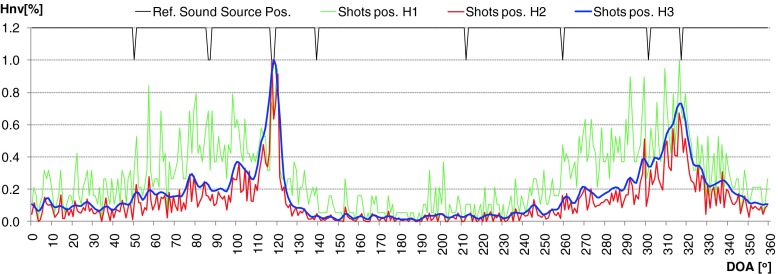
Normalized time distribution of DOA values for impulse sound sources obtained by means of particular method – two shot sounds emitted from different directions

**Fig. 8:**
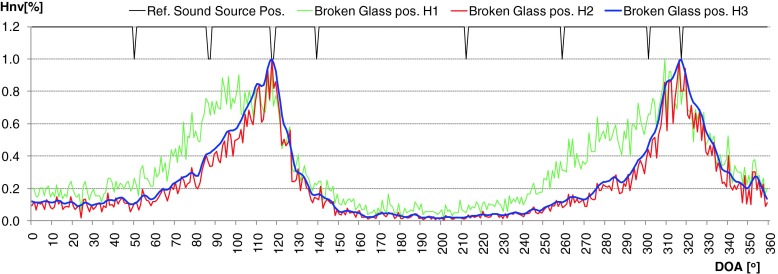
Normalized time distribution of DOA values for impulse sound sources obtained by means of particular method – two broken glass sounds emitted from different directions

**Fig. 9:**
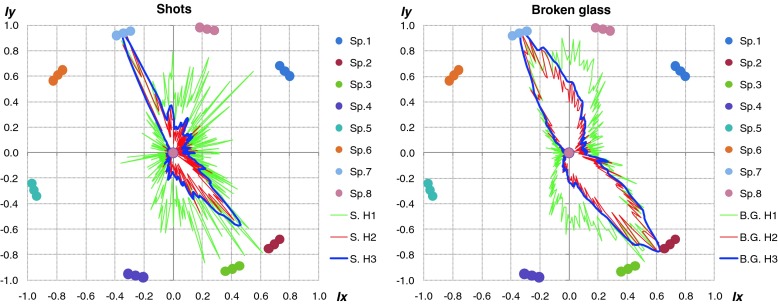
Polar representation of DOA values for considered method. *Left plot* – shot, *right plot* – broken glass. Increase of the localization accuracy for particular sound sources is clearly visible

During evaluation of the developed algorithm a several methods used to calculation time distribution of DOA were taken into consideration. First method (H1) assumed that the DOA values for particular direction was incremented by 1 if the frequency component in that direction was present. This approach worked well for signals whose time duration was longer than 0.5 [s]. For impulse sound sources (shot, broken glass) it was difficult to determine the main direction of arrival properly. In second method (H2), level of intensity vector for particular frequency of spectrum was taken into account. It means that the time distribution for given angle value indicate the total value of intensity vectors which occurred for given direction in considered time period. Thanks to this modification, the selectivity of obtained results increased rapidly. But for impulse sounds sources many additional local peaks were still observed. In some cases uncertainty of final decision about position of particular sound sources was high, because two or more local maximum were observed. Application of weighted moving average solved that problem (H3). Averaged period was equal to 7. Weighted coefficients were calculated based on Hanning window. In Figs. 7 and 8 normalized time distribution of DOA values (Hnv[%]) for impulse sound sources obtained by means of particular method were presented. In Fig. 9 the polar representation of the DOA values were shown.

The green line - H1 indicates the method 1, red line - H2 and blue line - H3, method 2 and 3 respectively. The black peaks indicate the reference position of the sound sources. If the black peak is common with the peak of the DOA it means that the position of sound source was indicated properly. Sound sources were simulated by speakers 2 and 6. In the left plot the localization results for shot sounds were depicted. Localization results for two broken glass played simultaneously were shown in right plot. It is clearly to notice that indication of localization for impulse sound sources for method H1 and H2 is ambiguous. Weighted moving average produce smooth DOA characteristic. Two main peaks can be observed. Automatic precise localization of the sound source position were possible in consider case.

### Length of overlap

**Fig. 10:**
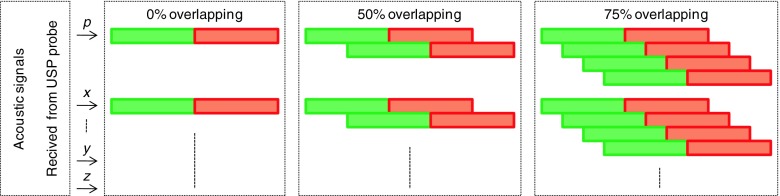
Tested overlapping configuration. *Green rectangles* – current frame, *red* – next frame

**Fig. 11:**
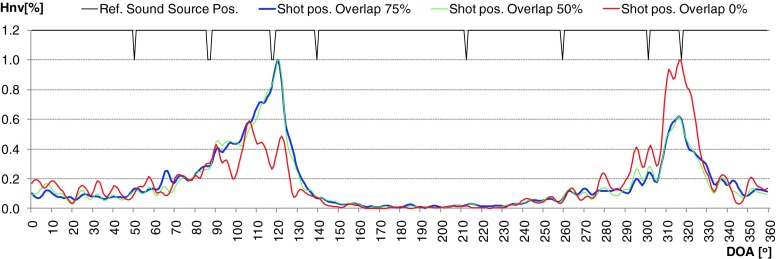
Time distribution of DOA values for different overlapping level for two shots presented at the same time

**Fig. 12:**
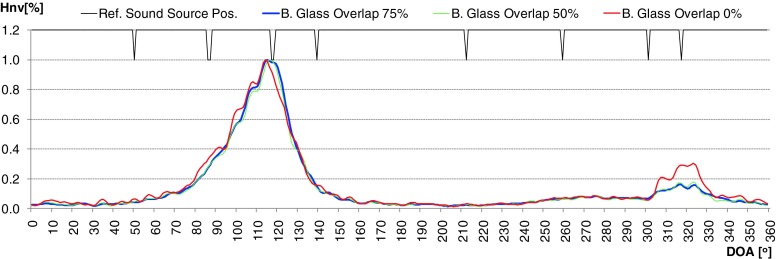
Time distribution of DOA values for different overlapping level for two broken glass presented at the same time

Another issue that was considered in evaluation process was the length of overlap used in the FFT calculations. Three different overlap values were tested: 0 %, 50 % and 75 %. It was explained in Fig. 10. Particular FFT frames were marked with different color. It means that in the next FFT frame from 4096 to 1024 samples were new. On the other hand for 4096 signal samples from 1 to 4 FFT frames were calculated. The FFT calculations were performed for each acoustic components separately. In Figs. 11 and 12 time distribution of DOA values for different overlapping lengths were presented. The results for impulse sounds like shots or broken glass were shown.

For longer acoustic events the difference was very low and it was not presented here. On the basis of obtained DOA characteristics optimal length of overlap was specified. It was equal to 50 %. Lower value can cause the loss of data, especially for impulse sounds. Such situation can be observed in Fig. 12.

On the other hand greater overlap value did not deliver more data during the time distribution calculation. High similarity of green and blue curves proved this statement. 50 % overlap was finally applied in the designed localization method.

### Localization results for synthetic signals

**Fig. 13:**
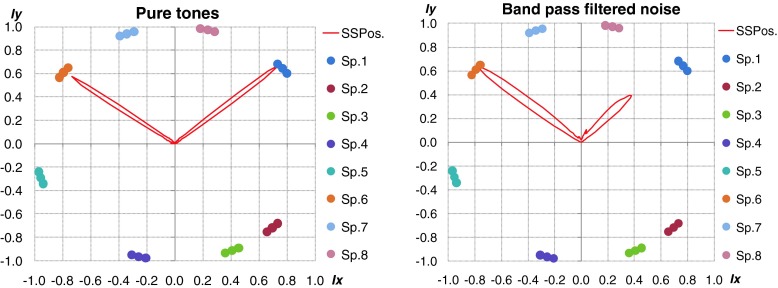
Localization results for synthetic signals. *Left plot* - pure tones 1000 Hz and 1250 Hz, *right plot* - 1/3 octave band filtered pink noise, the centre frequency 1000 Hz and 1250 Hz

The localization results for pure tones and 1/3 octave band pink noise were presented in Fig. 13. Test signals were emitted from loudspeaker 1 and 6.

For signals different in the frequency domain the localization accuracy was the best. Particular sound sources were localized perfectly. But when the identical, time synchronized, signals were presented from different directions, the proper localization of sound sources was insufficient and in some cases completely impossible.

### Localization results for real signals

**Fig. 14:**
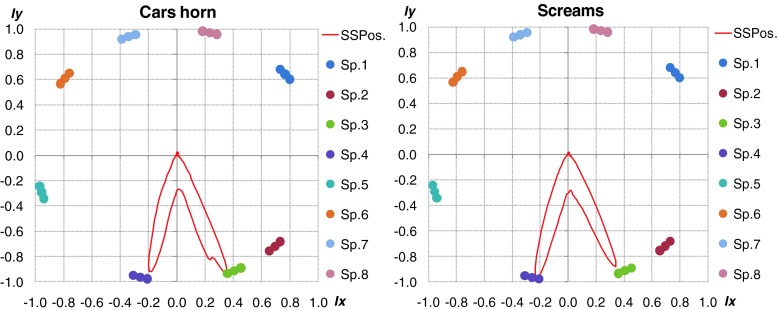
Localization results for real acoustic signals presented simultaneously by speakers 3 and 4. *Left plot* - car horn, *right plot* - scream

Results of localization accuracy obtained by means of real signals are presented below. In Fig. 14 car horn and scream were played from speaker 3 and 4. In this case the localization was precise for different signals. High inaccuracy was noticed for coherent sound sources.

**Fig. 15:**
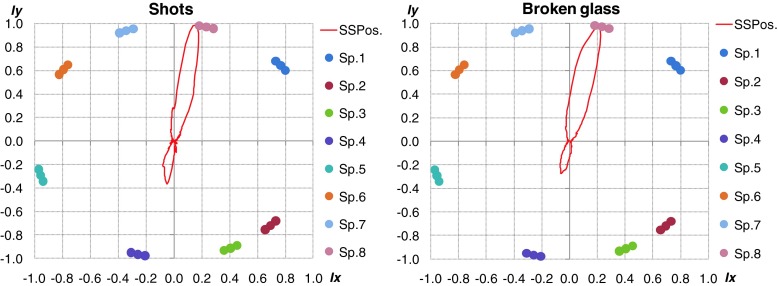
Localization results for real acoustic signals presented simultaneously by speakers 4 and 8. *Left plot* - shot, *right plot* - broken glass

**Fig. 16:**
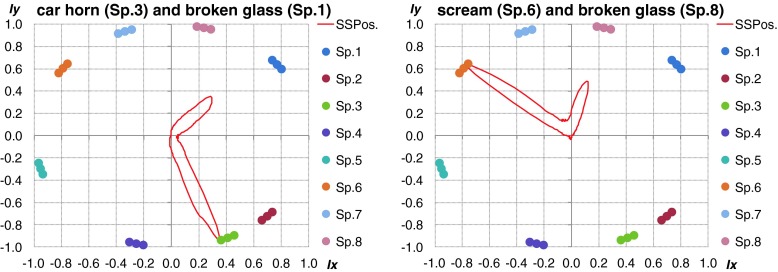
Localization results for different kind of sound sources presented simultaneously. *Left plot* – car horn and broken glass, *right plot* – scream and broken glass

Additionally, the localization accuracy goes down rapidly if sound sources were close to each other and amplitude one of them was dominant. It is important to emphasize, that for impulse sound sources the localization was based on few FFT frames (it depended on length of the considered acoustic event). Angular resolution of multiple sound sources localization also depended on the type of analyzed signal. Length of particular signals has crucial role for final accuracy. The best results were obtained for signals lasting more that 1 s. The type of given sound source is main difficulty for proper blind localization process in real time. If the analysis will be done offline during the situation reconstruction process, user can precisely select interest part of signal and do the localization process more accurate. Proposed algorithm can be used as an interesting and useful tool during the offline forensic audio analysis. In such case all of described parameters could be selected and changed manually adequate to considered signal. In Fig. 15 the localization results of impulse sound were shown. The localization was proper for signals different in frequency domain. In Fig. 16 localization results for different type of sound sources were shown.

### Localization accuracy for synthetic signals

In this section the detailed data of calculated localization accuracy results for synthetic signals are presented. The localization error was calculated according to formula 3 defined in Section 3 and was expressed in degrees. The complete results both for tonal signals and 1/3 octave band noise signals were presented in Table 3. Labels S1 and S2 indicate the signals that was played during the measurement session. For scenario no. 1 both signal has the same frequency. This case was indicated in the table by a grey color. When the signals were presented separately, their direction of arrival was indicated properly. During the simultaneous presentation of the same signals the localization results were insufficient. Only one sound source was observed and its position was between the real position of the sound sources. Second sound source was missing and it was marked in the table as ND (not detected). It means that for coherent signals (both in time and frequency domain) the proposed algorithm did not work properly. For different sound sources the localization accuracy obtained for simultaneous presentation of two sources was the same like for a single presentation. The similar accuracy results was obtained for tonal and noise signals.

**Table 3:** The localization accuracy results for tonal and 1/3 octave band noise signals

No.	S1	S2	Tonal signals				1/3 octave band noise signals			
			Single source		Two sources		Single source		Two sources	
	f [Hz]	f [Hz]	α_err S1_	α_err S2_	α_err S1_	α_err S2_	α_err S1_	α_err S2_	α_err S1_	α_err S2_
1	1000	1000	−2	12	ND	25	−10	3	ND	49
2	1000	250	−2	8	−2	8	−10	9	−10	9
3	1000	500	−2	10	−2	10	−10	5	−10	5
4	1000	800	−2	5	−2	5	−10	4	−10	5
5	1000	1250	−2	1	−2	1	−10	3	−10	2
6	1000	2000	−2	1	−2	0	−10	2	−10	2
7	1000	4000	−2	3	−2	3	−10	2	−10	2

### Localization accuracy for real signals

Tables 4, 5 and 6 include the localization accuracy obtained for real signals. Gray color indicate the situation when the same signal was presented through two different speakers. For this situation the high localization error was observed or only one sound source was indicated. Missing sound sources were marked in the tables as ND (not detected). In some cases (no. 15 scream) the localization was correct even for the same signals presented simultaneously from different directions. For all other measurement scenarios particular harmonic sound source was localized correctly. For these cases the localization accuracy was the same like for the presentation of particular signals independently. It means that the same type of sound source can be localized properly even if they were presented simultaneously.

**Table 4:** The localization accuracy results for signals: car horn and scream (harmonic signals)

No.	S1	S2	Car horn				Scream			
			Single source		Two sources		Single source		Two sources	
	α_GT_	α_GT_	α_err S1_	α_err S2_	α_err S1_	α_err S2_	α_err S1_	α_err S2_	α_err S1_	α_err S2_
1	40	197	−10	−6	−108	−19	−10	−5	−119	−13
2	314	110	7	4	5	3	5	2	5	2
3	294	40	5	−7	5	−7	5	−9	5	−8
4	255	294	−3	4	−3	3	−1	3	−2	3
5	197	143	−6	4	−4	4	−6	3	−6	2
6	143	77	2	−5	21	ND	3	−2	25	ND
7	110	314	4	3	4	3	2	4	1	5
8	77	255	−5	1	−6	1	−2	−1	−6	−2
9	40	143	−11	4	−11	5	−7	3	−8	3
10	314	77	3	−2	21	ND	4	−1	−24	ND
11	294	314	2	3	0	3	3	4	2	5
12	255	197	1	−5	1	−5	−1	−4	0	−4
13	197	110	−3	3	29	−74	−4	2	25	−49
14	143	40	4	−9	3	−9	2	−7	2	−6
15	110	294	0	2	−4	ND	2	4	2	1

**Table 5:** The localization accuracy results for signals: shot and broken glass (impulse signals)

No.	S1	S2	Shot				Broken glass			
			Single source		Two sources		Single source		Two sources	
	α_GT_	α_GT_	α_err S1_	α_err S2_	α_err S1_	α_err S2_	α_err S1_	α_err S2_	α_err S1_	α_err S2_
1	40	197	−8	−3	ND	−9	−7	−2	ND	−16
2	314	110	8	5	5	0	5	1	5	1
3	294	40	5	−8	−2	−5	4	−7	7	−11
4	255	294	−2	7	−6	ND	−2	7	−6	ND
5	197	143	−4	4	55	1	−4	5	−3	ND
6	143	77	3	−4	30	ND	5	−2	24	ND
7	110	314	2	5	−2	6	2	2	2	−1
8	77	255	−5	−3	−6	−6	−1	−2	−6	−2
9	40	143	−11	4	ND	4	−10	6	−12	ND
10	314	77	8	−1	20	ND	5	−6	19	ND
11	294	314	5	6	ND	11	3	7	ND	9
12	255	197	−2	−5	ND	−10	−1	−6	ND	−5
13	197	110	−7	3	35	ND	−7	4	42	ND
14	143	40	5	−10	ND	−15	9	−10	44	ND
15	110	294	2	3	3	ND	2	6	3	33

**Table 6:** The localization accuracy for mixed sound sources (harmonics and impulse sounds)

No.	S1	S2	Type of source		Single source		Two sources	
	α_GT_	α_GT_			α_err S1_	α_err S2_	α_err S1_	α_err S2_
1	40	197	car horn	scream	−10	−4	−10	−4
2	314	110	car horn	shot	3	8	2	3
3	294	40	car horn	broken glass	4	−5	4	−10
4	255	294	car horn	speech	−2	6	−2	6
5	197	143	scream	shot	−4	5	−4	6
6	143	77	scream	broken glass	3	−2	3	−1
7	110	314	scream	speech	2	6	2	6
8	40	143	shot	speech	−10	5	−10	5
9	314	255	broken glass	speech	7	−2	7	−2

Table 5 includes the results for impulsive sound sources. Such sounds were very difficult to localize when they were presented simultaneously. It is important to emphasize that the sound sources were synchronized precisely. We can observe the situation that often only one sound source was indicated properly. Such situation occurs for short time signals because the localization result of particular sound sources were calculated for few FFT frames (if the signal is 0.1 [s] length we have only 2 FFT frames). It means that the presented algorithm works properly with the harmonics sound sources (it is the property of the FFT analysis).

In the Table 6 the results for mixed type of sound sources were presented. In this measurement scenarios the harmonic signals were also presented together with impulse sounds. We can observe very high localization accuracy when the two sound sources were presented together. It was generally the same like for a particular single source presented separately. This scenario could be common for typical situations than could occur in a real life. Typically we can observe many different sound sources active at the same time. Based on the obtained results it was shown that the proposed algorithm could be useful in the localization of particular sound sources even they produce acoustic energy simultaneously.

## Conclusions

Method and preliminary results of multiple sound sources localization in a real time using the acoustic vector sensor were presented in this study. Term of the multiple sound sources in this research means that two sources produced the acoustic energy simultaneously from different directions. The several properties of the developed algorithm were discussed in details on the basis of specially prepared tests conducted in laboratory conditions. First was a selection of the best method for computation the time distribution of DOA values. Second was discussion about length of overlap. Hanning weighted moving average and 50 % length of overlap were optimal and gave the greater localization accuracy. The multiple sound sources localization can be done by means single acoustic vector sensor and sound intensity computation in frequency domain.

Localization accuracy and angular resolution depended on length of the analyzed signals and local differences both in time and frequency domain. The best results were obtained for signals longer than 1 s and different in time and frequency domain. For shorter signals the decrease of accuracy and angular resolution were observed. Moreover, the proposed algorithm did not work properly for coherent signals (both in time and frequency domain). The type of the given sound source is a main difficulty for proper blind localization process in the real time. In such case the information about the position of detected sound sources is presented immediately. Additional types of information about the sound source like beginning, end and length of activity can be also obtained and be presented.

Method can be applied to analysis both fixed or moving sound sources. Their trajectory can be tracked independently. The described method can be useful in a surveillance systems to monitor and visualize the acoustic field of specified region. The direction of arrival can be used to control the Pan-Tilt-Zoom (PTZ) camera to automatically pointing it towards the direction of the detected sound source.

It is important to emphasize that the proposed method can be used as an interesting and useful tool also during the offline forensic audio analysis. The described algorithm can be also used as a visualization technique called spectrogram direction of arrival. In such case all of described parameters could be selected and changed manually adequate to considered signal.

In future work the method will be examined in real disturbance conditions such as bank operating room. Additional improvements of functionality as spatial filtration into the defined direction and integration with other DSP method such as adaptive detection and automatic classification of sound events will also be implemented (for this reason the Hanning window was applied in FFT calculation). The comparison of the presented solution with traditional methods based on microphone arrays will be done.
